# Heads-up surgery with three-dimensional display devices for cataract surgeries

**DOI:** 10.5935/0004-2749.20210059

**Published:** 2025-02-02

**Authors:** José de Paula Barbosa, João Crispim Ribeiro, André Jucá Machado

**Affiliations:** 1 Grupo de Estudos e Pesquisa em Oftalmologia, Centro Universitário Christus, Fortaleza, CE, Brazil; 2 Tecnologia Minimamente Invasiva e Simulação na Área de Saúde, Centro Universitário Christus, Fortaleza, CE, Brazil; 3 Universidade Federal do Ceará, Fortaleza, CE, Brazil

Ophthalmologists widely use binocular microscopes (BM) for cataract surgeries. However, these devices have certain limitations in terms of educational approach and ergonomics^(1,2)^.

This study aims to review the “heads-up” cataract surgery and compare it with the traditional surgery using BM.

We utilized the Medline database to retrieve articles published between 2016 and 2020 and perform a review of the literature. The descriptors included “Cataract” AND “Heads-Up” OR “3D”.

The evaluated outcomes were categorized as follows: “Ergonomics and comfort”, “Surgical duration”, “Complications”, “Educational function”, and “Miscellaneous”.

A total of 196 anterior and posterior segment surgeries were performed, including three studies. The number of surgeries in the other two studies was not specified. Cataract surgeries were performed in 165 (84.2%) of the 196 eyes; the heads-up was used in 107 surgeries and BM in 58 surgeries (Table 1).

**Table 1:** Frequency of patients admitted for cataract surgeries and the main topics discussed in each study

Reference	Total N of eyes	N of cataract surgeries	N of cataract HUS	N of cataract BMS	Main topics discussed
2019 Qian^^2^^	20	20	10	10	Surgical duration; Complications; Educational function; Miscellaneous
2019 Eckardt^^3^^	Undefined	Undefined	Undefined	Undefined	Educational function
2019 Ohno^^4^^	Undefined	Undefined	Undefined	Undefined	Miscellaneous
2019 Matsumoto^^5^^	74	72	72	0	Miscellaneous
2020 Berquet^^1^^	102	73	25	48	Ergonomics and comfort; Surgical duration; Complications; Miscellaneous
Total^*^	196	165	107	58	

* the total was calculated based only in the articles which specified how many patients were operated.

N= number; HUS= heads-up surgery; BMS= binocular microscope surgery.

## Ergonomics and comfort

One study evaluated the fluency of the surgery, presence of low back pain, and visual comfort during the surgeries^(1)^. A questionnaire was applied after the procedures, and the perception of the physicians suggested that the fluency of the three-dimensional (3D) surgery was slightly better than that of the BM surgery, but without statistical significance (p=0.09). The participants had received a prior 4-week training for the heads-up system, during which some professionals had expressed discomfort with the 3D-glasses during the initial surgeries due to “3D-asthenopia”^(1)^.

This work also compared back pain complaints between surgeons utilizing the BM and the 3D-device; however, both showed similar results, with back pain symptoms being present in 12%-17% of all surgeries^(1)^.

## Surgical duration

One article reported 25% lower duration for the heads-up surgeries (16.44 min) when compared with BM surgeries (21.44 min)^(1)^. However, a selection bias was present as surgeons preferred to operate complicated patients with the BM. The isolated factors leading to a longer surgical duration were “status of the surgeon” and “intraoperative complications”, which probably occurred because of the selection bias^(1)^ (Table 2).

**Table 2:** Comparison of the surgical durations among the filtered studies

Surgical duration	HUS	BMS	p-value
2019 Qian^^2^^	8.30 ± 1.73 min	9.03 ± 1.47 min	>0.05
2020 Berquet^1^	16.44 ± 4.36 min	21.44 ± 7.50 min	0.007

HUS= heads-up surgery; BMS= binocular microscope surgery.

## Complications

In one study, no major intraoperative complications were identified for either type of surgery^(2)^. Nonetheless, another article reported that during the heads-up procedures, only one chemosis associated with a superficial traumatic corneal ulcer occurred, while the microscope group presented four complications (two chemosis, one zonular dialysis, and one posterior capsule rupture with sulcus implantation). However, this finding could be explained by the selection bias which disposed the higher risk patients in the BM group^(1)^.

The improvement in the corrected visual acuity and the decrease in the endothelial cellular density one month after the surgery were similar for both methods^(2)^.

## Educational function

A research group employed a smartphone to help a resident student during the operation; both the senior doctor and the resident were using 3D displays. Hence, the professor who was distantly located from the patient was able to precisely visualize the resident’s movements with the smartphone’s microphone and give him orientations through a Bluetooth earphone. As heads-up displays permit professors to be away from the observer microscope, this technique minimizes the dilemma of when to advise the students about improving the surgical maneuvers without giving the patient the sensation of being in a training environment^(3)^.

## Miscellaneous

Microscopes offer higher quality images when compared with the HD cameras utilized in heads-up surgeries. One study used an 8K camera for simulating cataract, anterior segment, and posterior segment surgeries, which provided a resolution comparable to that of microscopic visualization. Nevertheless, higher the resolution, the harder it is to adjust the focus; this raises the lighting necessary during the surgery to produce images with a high signal/noise ratio^(4)^.

It is also possible to amplify the lighting in the image by 23 times while utilizing the heads-up equipment, which consequently demands lower levels of light and avoids uncomfortable or harmful situations^(5)^.

Furthermore, a lag in the monitor was reported during the 3D surgery, mainly in the anterior segment maneuvers. However, it was irrelevant and did not prolong the procedure^(2)^.

Thus, the 3D surgeries did not show relevant disadvantages in terms of complications, surgical time, or musculoskeletal symptoms. Furthermore, 3D technologies enable significant educational improvements not only for the surgeons, residents, and students but also for the workers in the operation room as they permit enhanced comprehension of the surgical steps.
